# *MGMT* promoter methylation in triple negative breast cancer of the GeparSixto trial

**DOI:** 10.1371/journal.pone.0238021

**Published:** 2020-08-25

**Authors:** Paul Jank, Claire Gehlhaar, Lederer Bianca, Fontanella Caterina, Schneeweiss Andreas, Thomas Karn, Frederik Marmé, Hans-Peter Sinn, Marion van Mackelenbergh, Bruno Sinn, Dirk-Michael Zahm, Barbara Ingold-Heppner, Christian Schem, Elmar Stickeler, Peter A. Fasching, Valentina Nekljudova, Eliane Tabea Taube, Frank Heppner, Volkmar Müller, Carsten Denkert, Sibylle Loibl

**Affiliations:** 1 Institut für Pathologie, Philipps-Univeristät Marburg, Marburg, Germany; 2 Institut für Neuropathologie, Charité –Universitätsmedizin Berlin, Corporate Member of Freie Universität Berlin, Humboldt—Universität zu Berlin and Berlin Institut of Health, Berlin, Germany; 3 Lette Verein Berlin, Berlin, Germany; 4 German Breast Group Forschungs GmbH, Neu-Isenburg, Germany; 5 Department of Oncology, S. Martino Hospital, Belluno, Italy; 6 Nationales Centrum für Tumorerkrankungen, Universitätsklinikum und Deutsches Krebsforschungszentrum, Heidelberg, Germany; 7 Klinik für Frauenheilkunde und Geburtshilfe, Universitätsklinikum Frankfurt, Frankurt, Germany; 8 Universitätsfrauenklinik Mannheim, Mannheim, Germany; 9 Universitätsfrauenklinik Heidelberg, Heidelberg, Germany; 10 Universitätsklinikum Schleswig-Holstein, Klinik für Gynäkologie und Geburtshilfe, Schleswig-Holstein, Germany; 11 Institut für Pathologie, Charité –Universitätsmedizin Berlin, Corporate Member of Freie Universität Berlin, Humboldt—Universität zu Berlin and Berlin Institut of Health, Berlin, Germany; 12 SRH Wald-Klinikum Gera, Gera, Germany; 13 Institut für Pathologie, DRK Kliniken Berlin, Berlin, Germany; 14 Mammazentrum Hamburg, Hamburg, Germany; 15 Klinik für Gynäkologie, Uniklinik RWTH Aachen, Aachen, Germany; 16 University Hospital Erlangen, Erlangen, Germany; 17 Klinik für Gynäkologie, Universitätsklinikum Hamburg-Eppendorf, Hamburg, Germany; University of Kansas Medical Center and VA Medical Center, UNITED STATES

## Abstract

Triple-negative breast cancer (TNBC) is typically treated with chemotherapeutic agents, including carboplatin (Cb), an DNA platinating agent. The O6-methylguanine-DNA-methyltransferase gene (*MGMT*) encodes for the protein O6-alkylguanine-DNA-alkyltransferase (MGMT protein). MGMT protein is involved in DNA repair mechanisms to remove mutagenic and cytotoxic adducts from O6-guanine in DNA. In glioblastoma multiforme, *MGMT* methylation status is a predictive biomarker for increased response to temozolomide therapy. It has been suggested, that MGMT protein may have relevance for cellular adaptation and could have an influence on resistance to carboplatin therapy. We investigated the influence of *MGMT* promoter methylation on pathologic complete response and survival of patients with TNBC treated in the neoadjuvant GeparSixto trial. In 174 of 210 available TNBC tumors a valid *MGMT* promoter methylation status was determined by pyrosequencing of 5 CpG islands. In 21.8%, we detected a mean *MGMT* promoter methylation >10%. Overall, *MGMT* promoter methylation was not significantly associated with pathological complete response (pCR) rate. After stratification for the two therapy arms with and without Cb no statistically significant differences in therapy response rates between the two *MGMT* promoter methylation groups could be observed. Our results show that different *MGMT* promoter methylation status is not related to different chemotherapy response rates in the TNBC setting in GeparSixto.

## Introduction

Triple-negative breast cancer (TNBC) shows an aggressive clinical behavior, poor clinical outcome and has limited option for targeted therapies. Due to the absence of targets, like estrogen receptor (ER), progesterone receptor (PR) and growth factor receptors (HER2), TNBC does not benefit from hormonal or anti-HER2-based therapies and therapy strategies are focused on the use of chemotherapeutic agents, for example carboplatin [1].

Alterations in epigenetics, for example methylation status of CpG islands of DNA promoter regions, were considered as early and common events in cancer and play a major role in tumor progression [2–5]. Methylation is a special form of alkylation and the most common alkylating reaction in biology. Most DNA methylations in mammals take place at carbon-5 of the cytosine residues of cytosine-phosphatidyl-guanosine (CpG) dinucleotides and lead to a hypermethylation of DNA [5, 6]. Hypermethylation of CpG islands in gene promoter regions results in loss of the target protein, lack of mRNA expression or reduced enzyme activity [7–9].

The O6-methylguanine-DNA-methyltransferase gene (*MGMT*) encodes for the protein O6-alkylguanine-DNA-alkyltransferase (MGMT protein) and is located on chromosome 10q26. *MGMT* DNA repair activity of tumor cells is believed to contribute to resistance of tumor cells from cytotoxic effects of alkylating agents. MGMT protein is involved in DNA repair mechanisms to remove mutagenic and cytotoxic adducts from O6-guanine in DNA by transferring the alkyl group from the O6 position of guanine to a cysteine residue of its active side [10–16] and inactivating itself followed by ubiquitination [17]. Thereby, DNA-lesions caused by alkylating substances are repaired. Mutagenic adducts are caused by ionizing radiation, UV light, tobacco smoke or alkylating agents for example used in chemotherapy, such as carboplatin [10, 12]. Altogether, MGMT protein provides protection of normal cells from exogenous carcinogens and tumor cells from chemotherapeutic agents [18].

For glioblastoma multiforme, it has been shown that tumors with no or low levels of *MGMT* activity are more responsive to therapy with the alkylating drug temozolomide [19, 20]. *MGMT* inactivation by epigenetic silencing through methylation of CpG islands in the promoter region correlates with the sensitivity of tumor cells to alkylating agents. Therefore, at least in glioblastoma multiforme, *MGMT* methylation status is a predictive biomarker for increased response to temozolomide therapy [19].

In previous studies, Fumagalli et al. showed that different levels of *MGMT* methylation were present in TNBC, suggesting a biological function. In a cohort of 84 patients receiving different types of neoadjuvant chemotherapy no statistical significance regarding *MGMT* methylation and pathological complete response (pCR) or any other clinical pathological characteristics were observed [21, 22]. We extended this approach in a clinical trial setting investigating a neoadjuvant clinical trial cohort randomized to receive carboplatin therapy vs no carboplatin in addition to the anthracycline-taxane combination.

In this project, we investigated the hypothesis that *MGMT* promoter hypermethylation in TNBC is associated with an increased chemosensitivity and therefore an increased pCR rate. Furthermore, we hypothesized that *MGMT* methylated TNBC should benefit from adding DNA-damaging chemotherapeutics such as Carboplatin since the ability of DNA-repair is compromised.

We therefore evaluated the *MGMT* promoter methylation status by methylation-specific polymerase-chain reaction (PCR) and subsequent pyrosequencing of 5 CpG islands in TNBC core biopsies, from patients enrolled in a clinical phase II trial.

## Materials and methods

### Study population and histopathological examination

We retrospectively evaluated a cohort of TNBC tumors of patients enrolled in the GeparSixto (NCT01426880) trial [23] between August 2011 to December 2012. Patients gave written informed consent for trial participation and use of tissue for translational research. Ethics committee of Charité University Hospital Berlin approved use of human tissue for translational research project (EA1/139/05). GeparSixto is a phase II trial for investigation of adding carboplatin to a neoadjuvant chemotherapy (NACT) for patients with triple negative or HER2-positive primary breast cancer. Patients with stage II or III TNBC were randomized to receive 18 weeks of NACT treatment with paclitaxel (80mg/m^2^ q1w) and non-pegylated liposomal doxorubicin (Myocet, 20 mg/m^2^ q1w) + bevacizumab (15 mg/kg, q3w) with or without addition of carboplatin (AUC 2.0 q1w later reduced to AUC1.5 q1w per amendment after 330 patients). The relevant authorities and ethic committees approved this study including a translational program for which pretherapeutical formalin-fixed paraffin-embedded (FFPE) core biopsies were prospectively collected in the German Breast Group (GBG) tumor biobank. Hormone-receptor (HR) status (positive if >1% of ER/PR stained cells), HER2 status via immune-histochemically staining (IHC) (positive if IHC 3+ or IHC 2+ and silver *in-situ* hybridization [SISH]-positive), and Ki67 expression were centrally assessed prior to randomization.

### DNA preparation

FFPE core biopsy tissue with a tumor content ≥30% were cut as 3 x 5 μm into a reaction tube. For DNA preparation the slices were processed with QIAamp DNA-Mini-Kit from Qiagen GmbH (Hilden, Germany) according to manufacturer specifications. Genomic DNA was eluted in 20 μL PCR-water and concentration was determined using NanoDrop ND-1000 Spectrophotometer from Labtech International Ltd. (Ringmer, UK).

### Bilsulfite conversion

500 ng of each isolated genomic DNA (gDNA) were converted by using EZ DNA Methylation Kit™ from Zymo Research (Irvine, CA, USA). Except for the following step, the manufacturer instruction was used: 200 μL instead of 100 μL M-Wash Buffer were used before M-desulphonation.

### Pyrosequencing

The converted DNA was then amplified in a *MGMT* promoter specific PCR using the PyroMark Q24 System from Qiagen GmbH (Hilden, Germany). By adding 3 μL of converted DNA we followed the manufacturer instruction, but adapted the PCR cycling protocol (S1 Table).

After amplification, we followed the manufracturer instructions by using the PyroMark Vacuum Workstation and PyroMark Q24 System from Qiagen GmbH (Hilden, Germany) to detect methylated CpG islands in *MGMT* promoter DNA.

Results were determined by PyroMark Q24 software Qiagen GmbH (Hilden, Germany). Five CpG islands were measured and results reported as percentages. Out of five percent values we calculated a binary (*MGMT*bi) and a continuous (*MGMT*co) variable. For each CpG island the percentage of methylation was measured. In addition, the mean percentage of methylation across all CpG islands was calculated for each tumor. *MGMT* methylation was defined as positive if this mean value was >10%.

The continuous variable *MGMT*co (0–5) considered every single CpG island. The *MGMT*co value describes the number of CpG islands with a methylation of >10%.

### Statistical evaluation

For statistical analyses pathohistological data, *BRCA* mutation status and therapy response were used.

Pathological complete response (pCR) was defined as ypT0, ypN0. Disease-free survival (DFS) was defined as the time from random assignment until relapse (local, distant, or contralateral invasive breast cancer or secondary malignancies) or death irrespective of cause. Overall survival (OS) was defined as time from random assignment until death irrespective of the underlying cause. DFS and OS were analysed by the Kaplan–Meier method and log-rank tests.

Associations between binary variables were investigated with Fisher’s exact and between other categorical variables *χ*^2^ test.

## Results

### Tumor and patient characteristics

**Fig 1:**
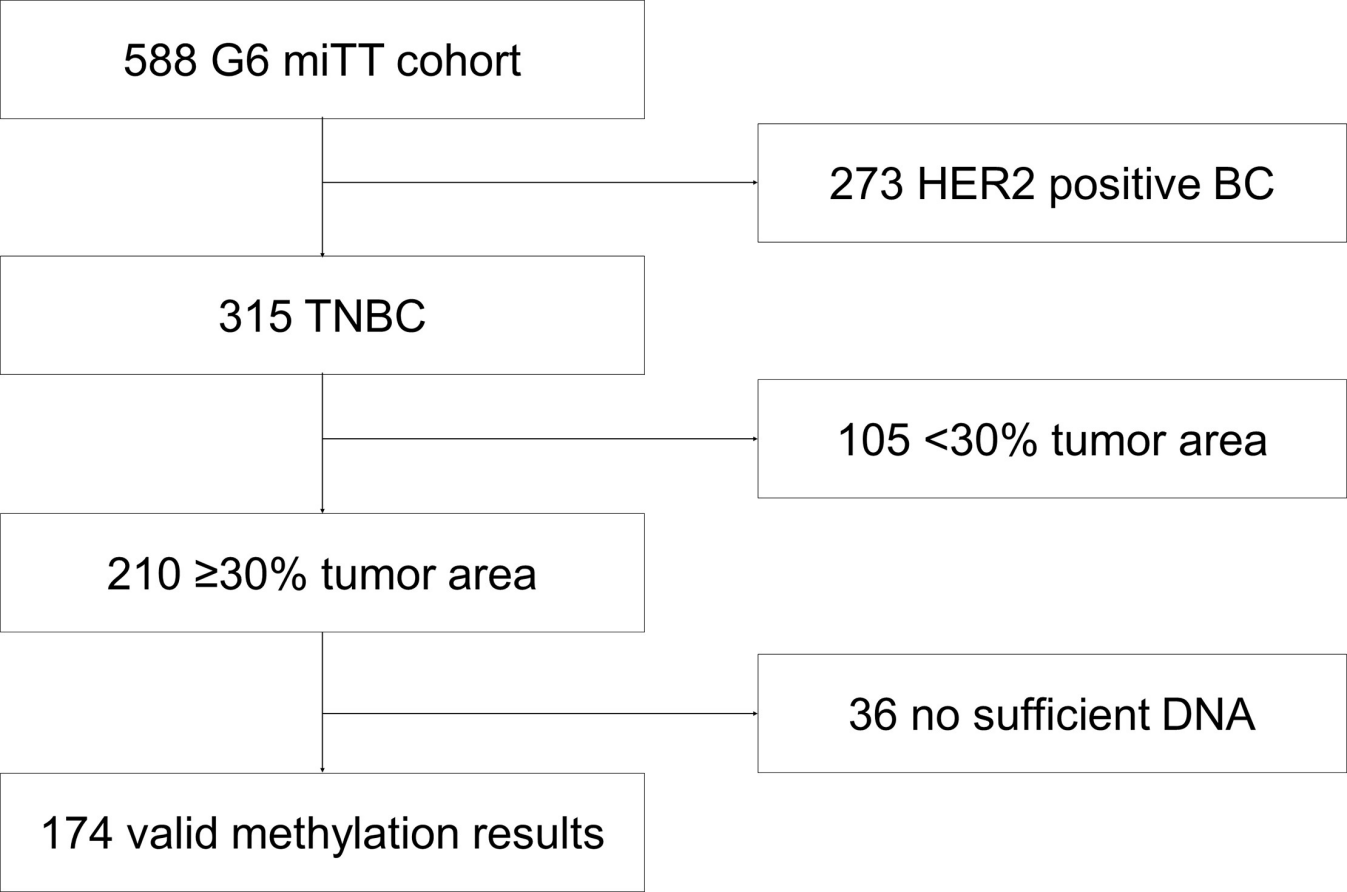
CONSORT diagram for *MGMT* promoter methylation trial in triple-negative (TNBC) GeparSixto (G6) cohort.

As shown in Fig 1, 210 pretherapeutic core biopsies, from a total of 315 triple-negative tumors, had sufficient remaining FFPE material with a tumor content ≥30% and were available for the methylation assay. In 174 (82.9%) tumors the *MGMT* methylation assay was performed successfully.

A *MGMT* promoter hypermethylation (*MGMT*bi) was observed in 38 (21.8%) tumors and a methylation mean ≤10% in 136 (78.2%) tumors, which were determined as not methylated (Table 1). Most BC were invasive-ductal (NST) breast carcinomas.

**Table 1:** Baseline characteristics in trial cohort.

		Overall N (valid %)	*MGMT* unmethylated N (valid %)	*MGMT* methylated N (valid %)	p-value
**N total**		174 (100.0)	136 (78.1)	38 (21.8)	
**Age**	**<50**	100 (57.5)	79 (58.1)	21 (55.3)	0.853
	**≥50**	74 (42.5)	57 (41.9)	17 (44.7)	
**Tumor staging**	**cT1**	53 (30.5)	38 (27.9)	15 (39.5)	0.231
	**cT2-4d**	121 (69.5)	98 (72.1)	23 (60.5)	
**Nodal status**	**cN0**	98 (56.3)	75 (57.3)	23 (60.5)	0.852
	**cN+ (≥1)**	71 (40.8)	56 (42.7)	15 (39.5)	
**Tumor grading**	**G1-2**	43 (24.7)	33 (24.3)	10 (26.3)	0.833
	**G3**	131 (75.3)	103 (75.7)	28 (73.7)	
**Ki-67**	**low (≤20%)**	10 (5.7)	9 (6.6)	1 (2.6)	0.693
	**high (>20%)**	164 (94.3)	127 (93.4)	37 (97.4)	
**Histology subtype**	**invasiv ductal (NST)**	162 (93.1)	128 (94.1)	34 (89.5)	0.298
	**invasiv lobular and other**	12 (6.9)	8 (5.9)	4 (10.5)	
**Tumor BRCA status**	**negative**	112 (64.4)	88 (79.3)	24 (77.4)	0.807
	**positive**	30 (17.2)	23 (20.7)	7 (22.6)	
**Pathological complete response**	**No pCR**	96 (55.2)	74 (54.4)	22 (57.9)	0.717
	**ypT0, ypN0**	78 (44.8)	62 (45.6)	16 (42.1)	

*MGMT* unmethylated: mean *MGMT* promoter methylation ≤ 10%; *MGMT* methylated: mean *MGMT* promoter methylation > 10%. Abbreviations: *BRCA*. Breast Cancer 1/2; NST, no special type; Ki-67, proliferation marker Ki-67 antibody; pCR, pathologic complete response; P-values refer to *MGMT* unmethylated vs. *MGMT* methylated. Percent values describes proportion of patients regarding to attributes for each group (overall, *MGMT* unmethylated, *MGMT* methylated).

Baseline characteristics, like Tumor staging, Nodal status, Grading and others factors were well balanced between the *MGMT* methylated and unmethylated subsets and shown in Table 1.

With respect to baseline characteristics, there were no differences between the GeparSixto trial cohort with and without known *MGMT* status, except for tumor grading and Ki-67 expression, as shown in S2 Table.

### *MGMT* promoter methylation in study cohort

According to both therapy arms, the *MGMT* promoter methylation status was distributed nearly identically. In the carboplatin arm, 24.3% of patients had a hypermethylated *MGMT* promoter, while in the arm without carboplatin 19.4% of patients had a hypermethylated *MGMT* promoter region.

**Fig 2:**
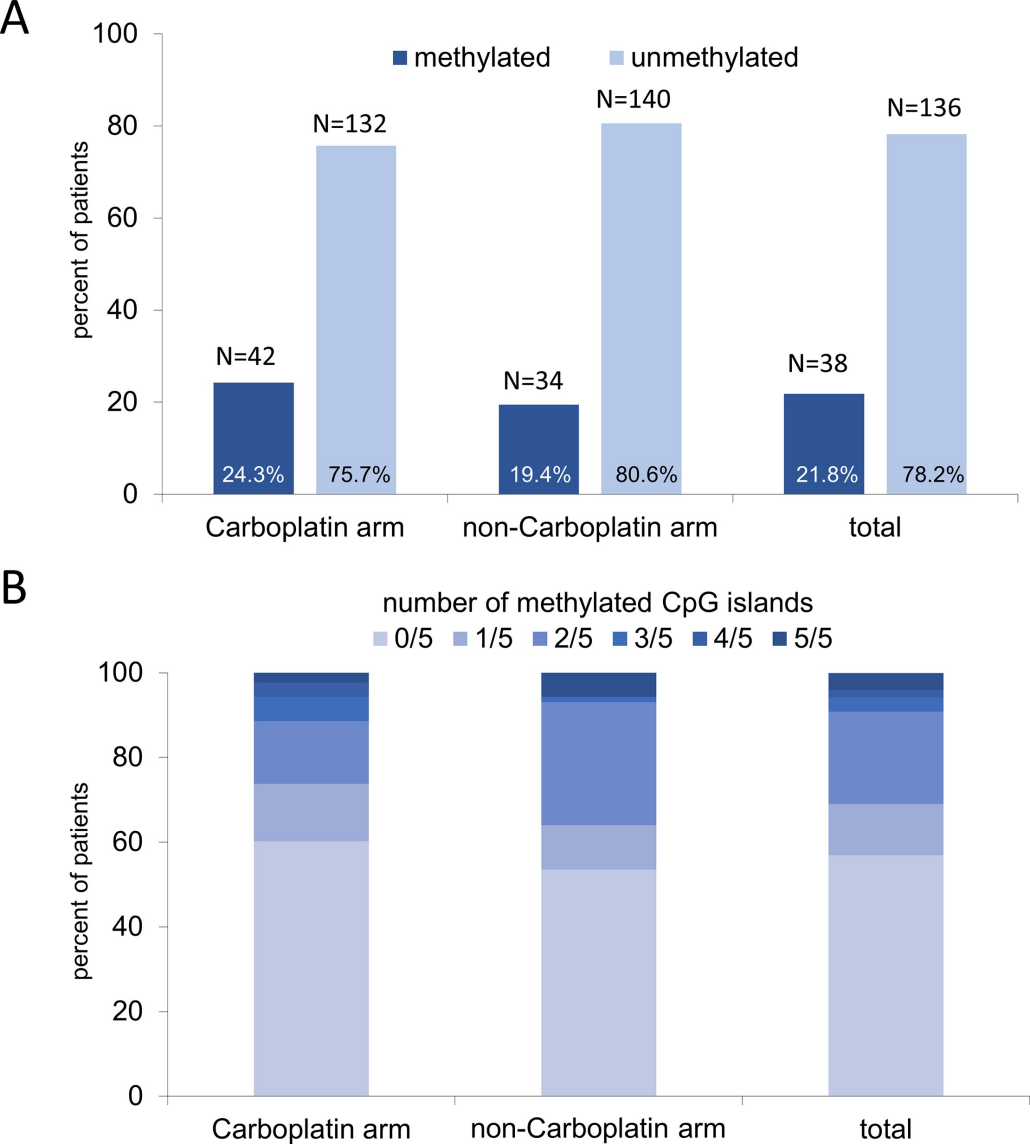
MGMT promoter methylation according to therapy regime in the GeparSixto trial, neoadjuvant treatment with and without addition of carboplatin. **A.** percent of patients with MGMT methylation status as binary variable (MGMTbi) **B.** percent of patients with MGMT methylation status as continuous variable (MGMTco).

Regarding the number of methylated CpG islands in the *MGMT* promoter, we observed mostly none or two methylated CpG islands. Patients in the non-carboplatin therapy arm had more often 2 of 5 methylated CpG island compared to the cohort with carboplatin (Fig 2).

### Treatment benefit according to *MGMT* promoter methylation

**Fig 3:**
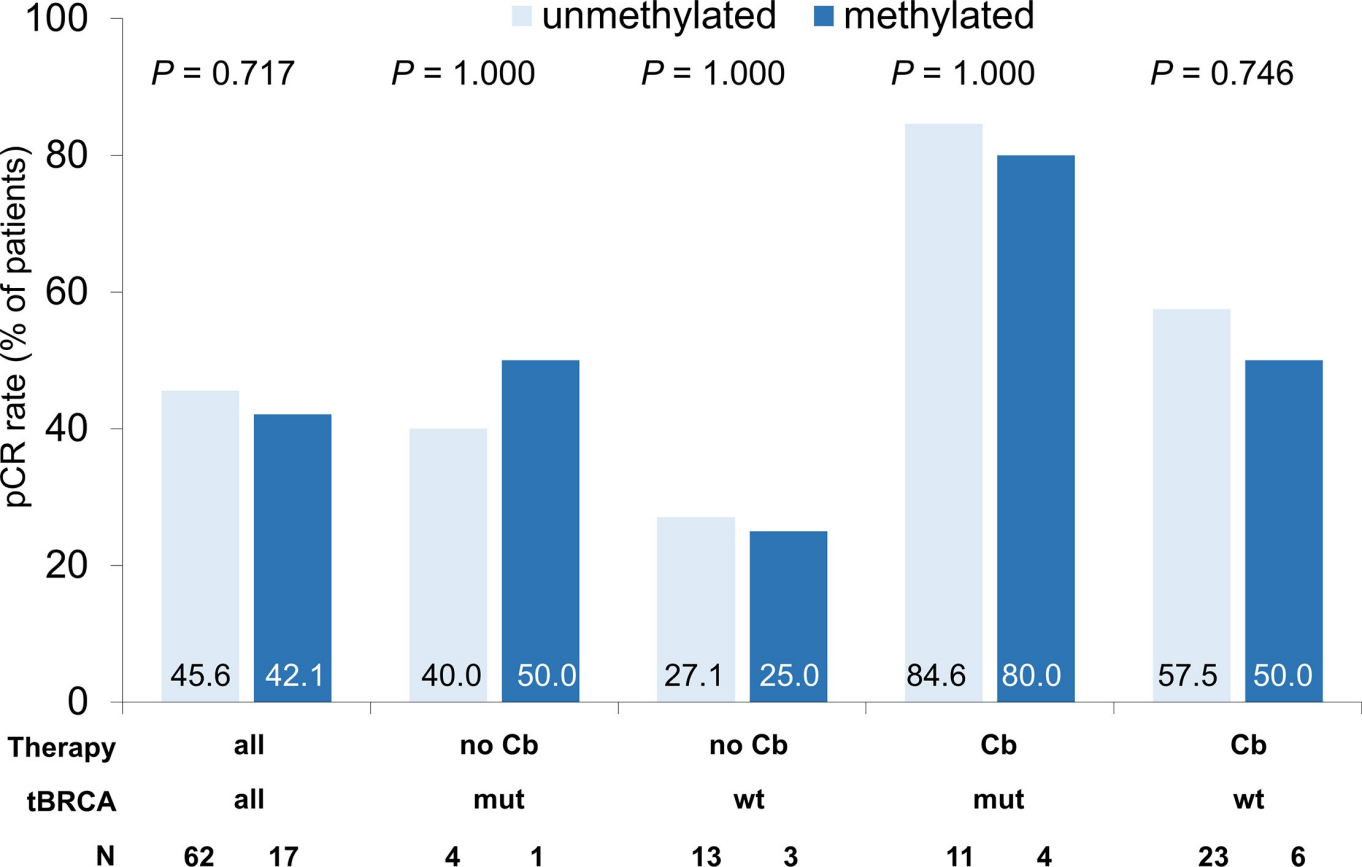
Pathological complete response (pCR) rate according to *MGMT* promoter methylation status (binary), therapy regime (with or without carboplatin) and *BRCA* mutation status. Therapy regime: no Cb = non-Carboplatin arm, Cb = Carboplatin arm. BRCA: wt = BRCA wildtype, mut = BRCA mutated. *P* values shown are calculated by Fisher‘s exact test.

Overall, *MGMT* promoter methylation was not significantly associated with pCR rate. The overall pCR rate was 44.8%, with no significant difference between the unmethylated subgroup (45.6%) and the methylated group (42.1%, *P* = 0.717; Fig 3).

Similar results were obtained for the different therapy arms. In the carboplatin arm, pCR rates were 84.6% for unmethylated *MGMT* and 80.0% for methylated *MGMT* for *BRCA* mutated tumors (N = 18, *P* = 1.000), 57.5% for unmethylated *MGMT* and 50.0% for methylated *MGMT* for *BRCA* wildtype patients (N = 52, *P* = 0.746).

In the non-carbo arm lower pCR rates were observed: pCR rates were 40.0% for unmethylated *MGMT* and 50.0% for methylated *MGMT* for *BRCA* mutated patients (N = 12, *P* = 1.000), 27.1% for unmethylated *MGMT* and 25.0% for methylated *MGMT* for *BRCA* wildtype patients (N = 60, *P* = 1.000).

### Survival analysis according to *MGMT* promoter methylation

**Fig 4:**
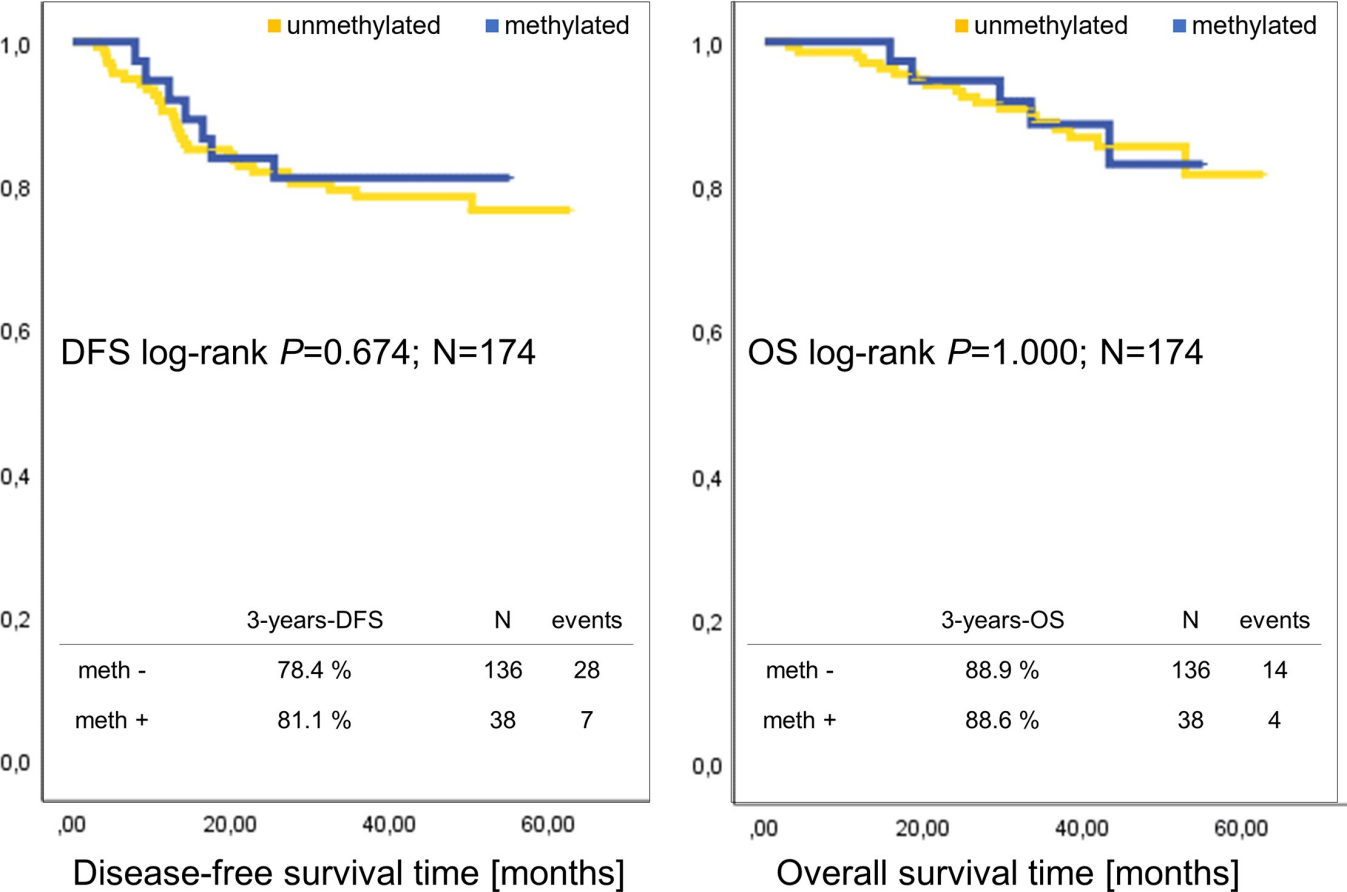
Kaplan-Meier analysis for comparison of prognosis of patients with *MGMT* promoter un-/methylated TNBC. DFS and OS time in months with binary *MGMT* promoter methylation status; p-values: log rank test.

In this cohort the median DFS and OS was not reached. We used the three years follow up (3Y-DFS, 3Y-OS) to determine possible differences: The 3Y-DFS in the *MGMT* promoter hypermethylated cohort is 81.1% and 3Y-OS = 88.6%, compared to the cohort with *MGMT* promoter unmethylated BC: 3Y-DFS = 78.4% and 3Y-OS is 88.6% (Fig 4). In addition, in S1 Fig we show the survival plots according to patients treated with and without the addition of Carboplatin.

## Discussion

We investigated the influence of *MGMT* promoter methylation on therapy response and survival of patients with TNBC, treated with chemotherapy with and without adding of carboplatin.

The role of *MGMT* promoter methylation for therapy response has been reported previously, especially for glioblastoma. In glioblastoma, an increased *MGMT* promoter methylation has a predictive impact for increased pCR and therapy response to temozolomide [19, 20].

For TNBC, the impact of *MGMT* promoter methylation status for therapy response has not been studied to a great extent in standardized clinical trial cohorts. To our knowledge, this is the first time *MGMT* promoter methylation was investigated in TNBC including a stratification by *BRCA* status and therapy regime.

We showed that *MGMT* promoter is hypermethylated in around 22% of TNBC, in this trial cohort. We did not observe any significant differences in age, tumor staging, nodal status, cell proliferation, therapy arm or histology type regarding *MGMT* promoter methylation status. Our study demonstrates that *MGMT* promoter methylation status has no predictive impact on pCR or outcome of patients with TNBC, neither overall nor stratified by carboplatin vs. control chemotherapy.

Fumagalli et al. have investigated *MGMT* methylation in an institutional cohort of 84 TNBC patients [22] and its role for response to neoadjuvant chemotherapy in patients treated with different agents [21]. Fumagalli reported higher *MGMT* promoter methylation prevalence, from 32.5% to 70%, with significant dependence on age. With respect to the age structure, both cohorts are different which might lead to different *MGMT* distributions. In both approaches, no correlation between clinicopathological data with *MGMT* methylation status were found. The results from Fumagalli et al. are the same as in our investigation.

Asiaf et al. investigated *MGMT* methylation and MGMT protein expression in a cohort of 123 invasive ductal BC (NST) and described a *MGMT* promoter methylation prevalence of 39.8% (51 of 128). Interestingly, they reached significant results for correlations of *MGMT* promoter methylation with clinicopathological markers such as tumor staging and grading and estrogen and progesterone receptor expression. Tumors with methylated *MGMT* promoter were more often stage II–IV with higher grading. Regarding hormone receptor expression, *MGMT* methylated tumors were more often HR (PgR or ER) negative [24]. Regarding tumor staging, our results show an opposite trend. Our cohort and the cohort from Asiaf et al. show differences in baseline parameters, which can explain this different results. In our cohort we only used TNBC and can not compare HR expression with MGMT methylation.

Isono et al. described a MGMT protein expression profile in different breast cancer subtypes: In luminal breast cancer 67% were MGMT high expressing (high), 46% in the TNBC cohort and 30% in the HER2 positive cohort, with a p-value of < 0.001 [25]. Regarding cell culture experiments, Bobustuc et al. observed the same: ER positive breast cancer cell lines expressed higher MGMT on protein level compared to ER negative cell lines [26]. The meta-analysis from An et al. shows a significant association of MGMT methylation with ER negative and grade 3 breast tumors [27]. Increased MGMT protein activity is associated with HR-positive breast cancer subtypes, while lower expression reduces MGMT protein activity. Increased methylation is associated with higher histological grade in basal-like tumors.

The strength of our investigation is that we used a prospectively collected clinical trial cohort with standardized treatment and available outcome data. As a limitation, due to the small size of pretherapeutic neoadjuvant core biopsies, it was not possible to perform a successful analysis in all patients that participated in the trial.

Results for glioblastoma were determined via MethyQESD method, described by Bettstetter et al. [28], using *MGMT* promoter methylation specific quantitative real-time PCR [20]. Bettstetter et al. performed a proof-of-principle, using survival data for cutoff-fitting. If more than 11.7% of *MGMT* gene promoter were methylated, the tumor was determined as *MGMT* promoter hypermethylated [28]. In our methylation assay, we used the common cutoff of 10 percent, determined via different methylation assays as previously described [20].

There are numerous other studies dealing with MGMT promoter methylation, MGMT mRNA expression and MGMT protein expression or MGMT protein activity in breast tumors and other diseases. Preuss et al. described a lack of MGMT protein activity in breast tumors of 5 percent [29]. An et al. described a strong association between MGMT promoter methylation and lower (or negative) MGMT protein expression (OR = 4.65, p<0.001) in breast cancer [27]. This association has been shown for other tumor types as well as on mRNA level [30]. Regarding glioblastoma MGMT promoter methylation seems to be the gold standard in clinical routine [31].

It should be noted that for prediction of pCR and outcome analysis other factors in addition to methylation of *MGMT* promoter or MGMT protein expression might be relevant. For a comprehensive analysis, additional relevant genes for DNA repair mechanism should be included [32]. Domagala et al. investigated pCR after neoadjuvant cisplatin therapy in a cohort of 43 tumors and the association with downregulation of DNA repair enzymes, such as *MGMT*, in *BRCA*1-associated TNBC. They showed, that DNA repair enzymes in patients with residual tumor are downregulated and may profit from PARP inhibitor therapy [32].

Interestingly, there are several studies investigating the use of temozolomide for breast cancer patients with brain metastases (NCT00617539, NCT00875355) as well as advanced or metastatic breast cancer without a history of brain metastases (NCT00194766, NCT00614978). Results are awaited soon.

Taken together, our results show that in the GeparSixto clinical trial cohort *MGMT* promoter methylation has no direct impact on therapy response to NACT with or without the addition of carboplatin in TNBC.

## Supporting information

S1 Table *MGMT* specific PCR profile. (TIF)

S2 Table Baseline characteristics in MGMT cohort compared to samples without MGMT status in GeparSixto trial. (TIF)

S1 Fig Kaplan-Meier analysis for comparison of prognosis of patients treated with and without Carboplatin. DFS and OS time in months with binary Carboplatin therapy regime; p-values: log rank test.
(TIF)
