# Correction: *MGMT* promoter methylation in triple negative breast cancer of the GeparSixto trial

**DOI:** 10.1371/journal.pone.0257142

**Published:** 2021-09-01

**Authors:** Paul Jank, Claire Gehlhaar, Bianca Lederer, Caterina Fontanella, Andreas Schneeweiss, Thomas Karn, Frederik Marmé, Hans-Peter Sinn, Marion van Mackelenbergh, Bruno Sinn, Dirk-Michael Zahm, Barbara Ingold-Heppner, Christian Schem, Elmar Stickeler, Peter A. Fasching, Valentina Nekljudova, Eliane Tabea Taube, Frank Heppner, Volkmar Müller, Carsten Denkert, Sibylle Loibl

The third, fourth, and fifth authors’ names appear incorrectly. The correct names are: Bianca Lederer, Caterina Fontanella, and Andreas Schneeweiss. The correct citation is: Jank P, Gehlhaar C, Lederer B, Fontanella C, Schneeweiss A, Karn T, et al. (2020) *MGMT* promoter methylation in triple negative breast cancer of the GeparSixto trial. PLoS ONE 15(8): e0238021. https://doi.org/10.1371/journal.pone.0238021

## References

[1] Jank P, Gehlhaar C, Bianca L, Caterina F, Andreas S, Karn T, et al. (2020) *MGMT* promoter methylation in triple negative breast cancer of the GeparSixto trial. PLoS ONE 15(8): e0238021. 10.1371/journal.pone.0238021 32841306 PMC7446962

