# Supplementary figures and images for: MGMT promoter methylation in triple negative breast cancer of the GeparSixto trial
Source: PLoS One. 2020 Aug 25;15(8):e0238021. doi: 10.1371/journal.pone.0238021 (PMC7446962; doi:10.1371/journal.pone.0238021)

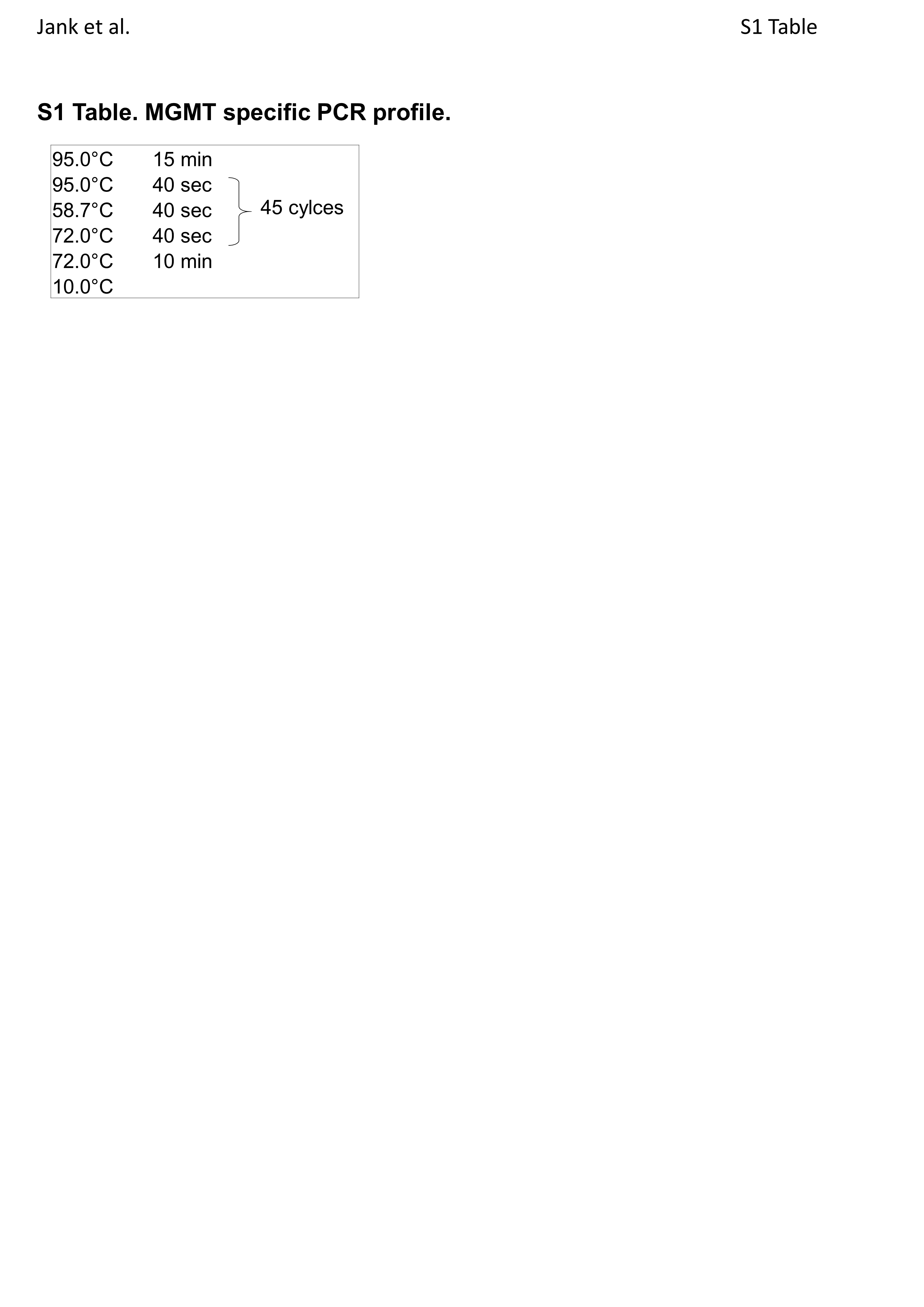

Supplement: S1 Table — (TIF) [file pone.0238021.s001.tif]

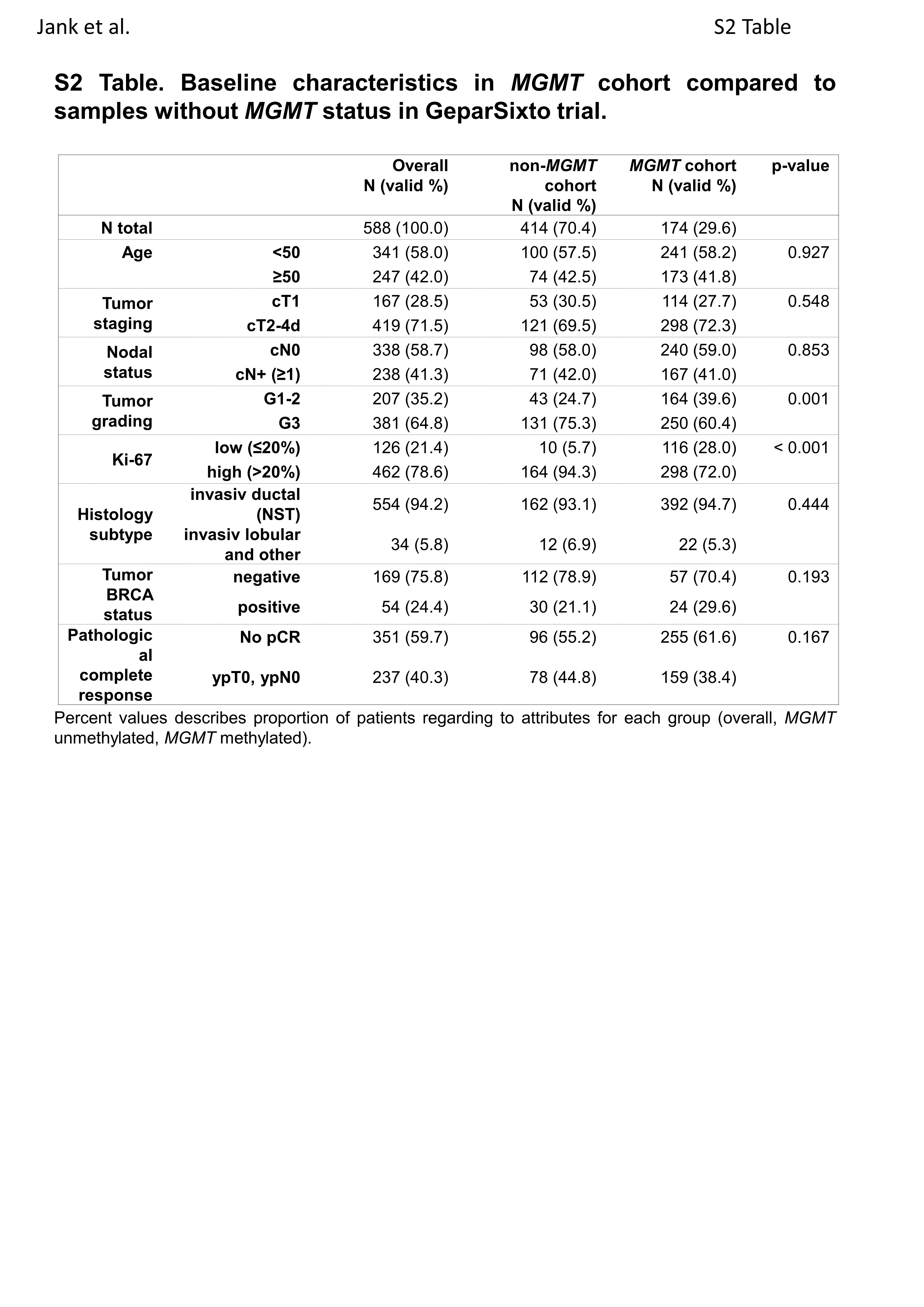

Supplement: S2 Table — (TIF) [file pone.0238021.s002.tif]

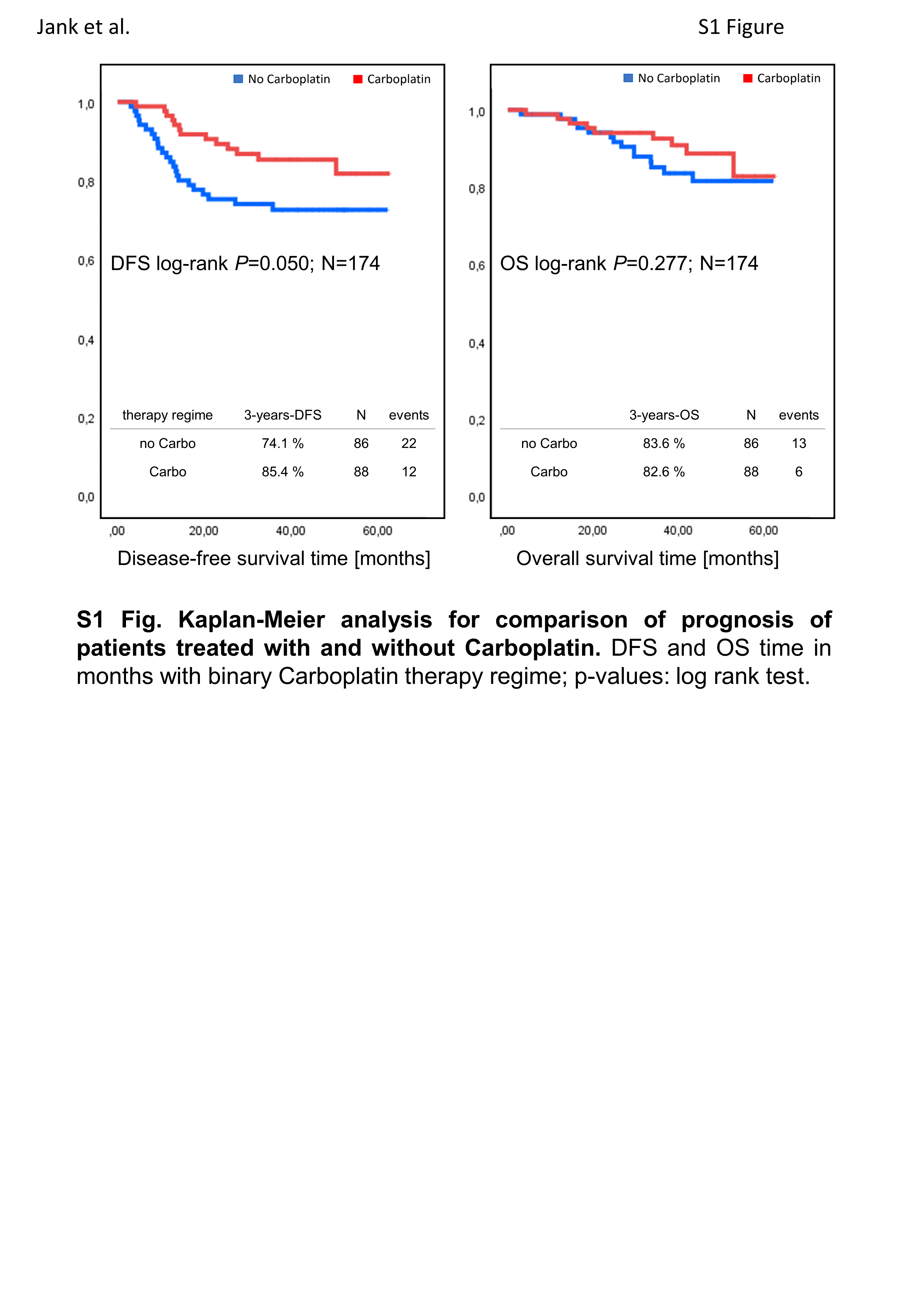

Supplement: S1 Fig — DFS and OS time in months with binary Carboplatin therapy regime; p-values: log rank test. (TIF) [file pone.0238021.s003.tif]
